# The Transferrin Receptor CD71 Delineates Functionally Distinct Airway Macrophage Subsets during Idiopathic Pulmonary Fibrosis

**DOI:** 10.1164/rccm.201809-1775OC

**Published:** 2019-07-15

**Authors:** Sarah J. Allden, Patricia P. Ogger, Poonam Ghai, Peter McErlean, Richard Hewitt, Richard Toshner, Simone A. Walker, Peter Saunders, Shaun Kingston, Philip L. Molyneaux, Toby M. Maher, Clare M. Lloyd, Adam J. Byrne

**Affiliations:** ^1^ Inflammation, Repair, and Development Section, National Heart and Lung Institute, Faculty of Medicine, Imperial College, London, United Kingdom; ^2^ UCB Celltech, Slough, United Kingdom; and; ^3^ NIHR Respiratory Biomedical Research Unit, Royal Brompton Hospital, London, United Kingdom

**Keywords:** airway macrophages, idiopathic pulmonary fibrosis, transferrin receptor

## Abstract

**Rationale:** Idiopathic pulmonary fibrosis (IPF) is a devastating progressive disease with limited therapeutic options. Airway macrophages (AMs) are key components of the defense of the airways and are implicated in the pathogenesis of IPF. Alterations in iron metabolism have been described during fibrotic lung disease and in murine models of lung fibrosis. However, the role of transferrin receptor 1 (CD71)-expressing AMs in IPF is not known.

**Objectives:** To assess the role of CD71-expressing AMs in the IPF lung.

**Methods:** We used multiparametric flow cytometry, gene expression analysis, and phagocytosis/transferrin uptake assays to delineate the role of AMs expressing or lacking CD71 in the BAL of patients with IPF and of healthy control subjects.

**Measurements and Main Results:** There was a distinct increase in proportions of AMs lacking CD71 in patients with IPF compared with healthy control subjects. Concentrations of BAL transferrin were enhanced in IPF-BAL, and furthermore, CD71^−^ AMs had an impaired ability to sequester transferrin. CD71^+^ and CD71^−^ AMs were phenotypically, functionally, and transcriptionally distinct, with CD71^−^ AMs characterized by reduced expression of markers of macrophage maturity, impaired phagocytosis, and enhanced expression of profibrotic genes. Importantly, proportions of AMs lacking CD71 were independently associated with worse survival, underlining the importance of this population in IPF and as a potential therapeutic target.

**Conclusions:** Taken together, these data highlight how CD71 delineates AM subsets that play distinct roles in IPF and furthermore show that CD71^−^ AMs may be an important pathogenic component of fibrotic lung disease.

**At a Glance Commentary**

**Scientific Knowledge on the Subject**

Alterations in iron metabolism have been described during fibrotic lung disease and in murine models of lung fibrosis. However, how the role of transferrin receptor 1 (CD71)-expressing airway macrophages contributes to the pathogenesis of idiopathic pulmonary fibrosis is not known.

**What This Study Adds to the Field**

We found that there was an increase in proportions of airway macrophages lacking CD71 in patients with idiopathic pulmonary fibrosis compared with healthy control subjects. CD71^−^ airway macrophages were characterized by reduced expression of markers of macrophage maturity, impaired phagocytosis, and enhanced expression of profibrotic genes. Proportions of airway macrophages lacking CD71 were independently associated with worse survival, underlining the importance of this population in idiopathic pulmonary fibrosis and as a potential therapeutic target.

Idiopathic pulmonary fibrosis (IPF) is a devastating disease with an unknown etiology characterized by deposition of excess extracellular matrix (ECM) and the destruction of lung architecture leading to compromised gas exchange (1). IPF is the most common form of interstitial lung disease (ILD), with more than 5,000 new cases diagnosed each year in the United Kingdom (2). Treatment options are limited for IPF, and existing therapies slow but do not reverse disease progression (2). There is therefore an urgent need to understand the fundamental mechanisms involved in IPF to devise new therapeutic approaches.

Airway macrophages (AMs) play essential roles in the maintenance of normal homeostasis of the airway and in host defense, repair, surfactant processing, and inflammatory cascades (3). In particular AMs have been shown to be important drivers of pulmonary fibrosis (4). Macrophages are strategically positioned at the interface between the airways and the environment and are found in close proximity to collagen-producing myofibroblasts, and they secrete numerous profibrotic soluble mediators, chemokines, and MMPs (matrix metalloproteases) (4). AMs in particular have been shown to be involved in the regulation of the ECM via secretion of MMPs or by direct uptake of collagen (5, 6).

Iron is an essential nutrient for numerous cellular processes; however, excess iron can also be harmful because it acts as a catalyst in the formation of free radicals from reactive oxygen species via the Fenton reaction (7). Iron content is therefore tightly regulated at the intra- and extracellular levels. In addition to limiting toxicity from iron overload, regulation of free iron serves as an innate immune mechanism against invading pathogens, limiting the availability for use by pathogenic microorganisms (8–10). Alterations in iron metabolism have been described in chronic lung diseases such as chronic obstructive pulmonary disease (8, 11), asthma (12–14), and IPF (15–17). Transferrin receptor 1, also known as CD71, is an integral membrane protein that mediates the uptake of diferric transferrin complexes via receptor-mediated endocytosis (18). At steady state, the majority of iron in the circulation is bound to transferrin, which limits iron-catalyzed free radical production and facilitates delivery to target cells (19). CD71 has been shown to be highly expressed on human AMs in multiple contexts; alterations in numbers of CD71-expressing AMs have been reported in sarcoidosis and hypersensitivity pneumonitis (20), as well as in ILD cohorts (21). CD71 expression has also been shown to be induced in AMs in murine models of lung fibrosis (22). However, the contribution of CD71-expressing AMs to the development of IPF is not known.

In this study, we sought to assess the role of CD71-expressing AMs in IPF. We found that in IPF airways, there was a distinct increase in proportions of AMs lacking CD71 compared with healthy control airways. The concentration of BAL transferrin was enhanced in IPF BAL, and furthermore, CD71^−^ AMs had an impaired ability to take up transferrin. CD71^+^ and CD71^−^ AMs were defined by clear differences in phenotype, function, and expression of profibrotic genes. Compared with CD71^+^ macrophages, AMs lacking surface expression of CD71 were characterized by reduced expression of markers of macrophage maturity, impaired phagocytosis, and enhanced expression of profibrotic genes. Importantly, CD71 AM status was associated with shorter survival in patients with IPF, highlighting this pathway as a potential prognostic factor or therapeutic target. Some of the results of these studies have been reported previously in the form of an abstract (23).

## Methods

### Collection of BAL Samples

All patients and control subjects provided written informed consent to participate in the study, which was approved by a Royal Brompton Hospital ethics committee (10/HO720/12). Bronchoscopies were performed with subjects under a light sedation with midazolam in combination with local anesthesia with lidocaine. Four 60-ml aliquots of warmed sterile saline were instilled in the right middle lung lobe and aspirated by syringe, and lavage aliquots collected after each instillation were pooled for each patient. Volume and BAL appearance were recorded for all samples.

### BAL Processing

BAL samples were processed and stained on the day of sample collection. Whole BAL was strained through a 70-μm sterile strainer and subsequently centrifuged (700 × *g*; 5 min; 4°C), and pellets were subjected to red blood cell lysis (155 mM NH_4_Cl, 10 mM KHCO_3_, 0.1 mM ethylenediaminetetraacetic acid, pH 7.3) for 10 minutes before washing and resuspension in complete media (RPMI with 10% fetal calf serum, 2 mM l-glutamine, 100 U/ml penicillin-streptomycin). Cells were counted and pelleted onto glass slides by cytocentrifugation (5 × 10^4^ cells/slide). Differential cell counts were performed by Wright-Giemsa–stained cytospin. Percentages of eosinophils, lymphocytes/mononuclear cells, neutrophils, and macrophages were determined from a total of 400 cells. To obtain absolute numbers of each leukocyte subtype, these percentages were multiplied by the total number of cells obtained in the lavage fluid.

### Flow Cytometry

Cells were washed and incubated with near-infrared fixable live/dead stain (Life Technologies Inc.) as per the manufacturer’s instructions. Cells were washed before incubation with human Fc block (BD Pharmingen, Inc.), and surface staining was performed with the following antibodies (fluorophore followed by clone in parentheses) for the analysis of AMs in BAL: CD45 (PE [phycoerythrin]-Cy5 [cyanine 5]; HI30), CD11c (PE-Cy7; 3.9), CD14 (BV711; M5E2), CD71 (PE; CY1G4), CD86 (PE-Dazzle; IT2.2), CD163 (APC [allophycocyanin]; GHI/61), CD206 (FITC [fluorescein isothiocyanate]; 15-2), MARCO (macrophage receptor with collagenous structure; APC; PLK-1 [Polo-like kinase 1]), SR-A (scavenger receptor A; BV421; U23-56), and HLA-DR (BV421; L243). Monocyte and dendritic cell (DC) populations were analyzed with the following panel: lineage cocktail comprised of CD3 (FITC; HIT3a), CD4 (FITC; OKT4), CD8 (FITC; HIT8a), CD19 (FITC; HIB19), CD20 (FITC; 2H7), CD34 (FITC; 561), FcεRI (Fcε receptor I; FITC; AER-37); CD45 (BV605; HI30), CX3CR1 (2A9-1), CCR2 (K036C2), CD36 (5-271), CD11b (AF700; M1/70), CD11c (PE-Cy7; 3.9), CD16 (peridinin chlorophyll protein-Cy5; B73.1), CD163 (APC; GHI/61), CD14 (BV711; M5E2), and CD71 (PE; CY1G4). All antibodies were purchased from BioLegend, Inc., with the exception of HLA-DR (BD Biosciences), SR-A (BD Biosciences), and MARCO (Thermo Fisher). Surface staining was followed by fixation and then permeabilization to allow for intracellular or intranuclear staining. For transferrin uptake and nitric oxide assays, cells were plated at 1 × 10^5^ per well and resuspended in PBS containing either 5 μM transferrin (catalog no. P35376; Life Technologies) or 0.1 μM nitric oxide stain (4-amino-5-methylamino-2′,7′-difluororescein diacetate, catalog no. D23842; Life Technologies) or PBS alone for 30 minutes at 37°C, 5% CO_2_, and subsequently stained for flow cytometry. For phagocytosis assay, 2.5 × 10^5^ cells were resuspended in 200 μl of complete RPMI (RPMI 1620; catalog no. 21870-076, 10% FBS, 1,000 U/ml penicillin, and 1 mg/ml streptomycin; Life Technologies) alone or containing 0.125 mg/ml *Staphylococcus aureus* bioparticles (catalog no. P35367; Life Technologies) for 2 hours. Cells were then washed with PBS and stained for flow cytometry. Labeled cells were acquired on a BD LSR Fortessa II fluorescence-activated cell sorter (BD Biosciences). Before acquisition of any data, photomultiplier tube voltages were calibrated to the highest signal-to-background ratio using the BD Cytometer Setup and Tracking Beads Kit (catalog no. 655051; BD Biosciences). Then, using single stained compensation beads (UltraComp eBeads; Thermo Fisher) and the automated compensation program available in BD FACSDiva software (BD Biosciences), a compensation matrix was created and directly applied to every subsequent sample. Data were exported and further analyzed by using FlowJo software.

### Immunofluorescence Staining and Imaging

Cell surface staining was performed using a primary CD71 antibody (catalog no. PA5-27739; Sigma-Aldrich) or negative control antibody (rabbit immunoglobulin fraction, catalog no. X0936; Dako) followed by goat antirabbit Alexa Fluor 488 (catalog no. A-11008; Life Technologies) and counterstained with DAPI (catalog no. P36941; Life Technologies). To visually confirm/image phagocytosis, 1 × 10^5^ whole BAL cells were placed in an 8-well chamber slide (catalog no. 734-2050; VWR) in live cell imaging solution containing 1% bovine serum albumin (catalog no. A14291DJ; Life Technologies). Bacterial phagocytosis was captured 2 hours after addition of 100 μl of 0.25 mg/ml pHrodo Green *S. aureus* BioParticles (catalog no. P35382; Life Technologies) for 2 hours at 37°C, 5% CO_2_, with Hoechst 33342 nuclear stain (catalog no. R37605; Life Technologies) and imaged using a Leica DM2500 microscope (Leica Microsystems), and data were analyzed using ImageJ-win32 software. Staining for iron in cytospin preparations was performed using a Prussian blue test kit (catalog no. HT20; Sigma-Aldrich).

### Real-Time PCR

RNA extraction was performed using the RNeasy Plus Micro Kit (Qiagen) according to the manufacturer’s protocol. Total RNA was reverse transcribed into cDNA using the High-Capacity cDNA Reverse Transcription Kit (Thermo Fisher Scientific) as per the manufacturer’s instructions. Real-time PCR reactions were performed using Fast qPCR Master Mix (Thermo Fisher) on a ViiA 7 instrument (Life Technologies) with TaqMan primer sets (Thermo Fisher) for the following genes (catalog numbers in parentheses): *GAPDH* (Hs02758991_g1), *TLR1* (Toll-like receptor 1; Hs00413978_m1), *TLR2* (Hs02621280_s1), *TLR3* (Hs01551078_m1), *TLR4* (Hs00152939_m1), *TLR6* (Hs01039989_s1), *TLR7* (Hs01933259_s1), *TLR9* (Hs00370913_s1), *FcγRIA* (Hs00174081_m1), *ELMO1* (engulfment and cell motility protein 1; Hs00404992_m1), *CD71* (Hs00951083_m1), *ACO1* (aconitase 1; Hs00158095_m1), *IREB2* (iron-responsive element-binding 2; Hs00386293_m1), *SLC40A1* (solute carrier family 40 member 1; Hs00205888_m1), *SLC11A2* (solute carrier family 11 member 2; Hs00167206_m1), *HAMP* (hepcidin; Hs00221783_m1), and *HMOX* (heme oxygenase; Hs01558390_m1). Gene expression was analyzed using the change-in-threshold comparative cycle threshold method. Fold changes in mRNA expression for targeted genes were calculated relative to healthy controls. Examination of genes using the human fibrosis RT^2^ Profiler PCR Array (PAHS-120Z; Qiagen) was conducted following the manufacturer’s guidelines. All gene expression analysis was performed using RT^2^ Profiler PCR Array Data Analysis version 3.5 (Qiagen).

### Detection of Iron and Transferrin in BAL Samples

Concentrations of iron and related proteins were measured by using spectrophotometry kits for the detection of iron (catalog no. ab83366, Abcam; or catalog no. MAK025, Sigma-Aldrich) and ELISA kits for the detection of transferrin (catalog no. ab187391; Abcam) and ferritin (catalog no. ab108837; Abcam).

### Statistics

Differences between noncontinuous groups were compared using the Mann-Whitney *U* test. Spearman’s rank correlation was used to assess the association between continuous variables. Kaplan-Meier analysis was used to compare time to all-cause mortality between subjects dichotomized above and below median percentage CD71^−^. Multivariable Cox proportional regression was used to assess the association between CD71^−^ (expressed as a continuous variable) and plausible confounding variables (age, sex, baseline FVC percent predicted, and baseline Dl_CO_ percent predicted) with all-cause mortality. A backward stepwise selection procedure was used with each variable remaining in the model if *P* < 0.10. Because of variations in sample recovery and cell numbers available for experiments, *n* values differ between some readouts; sample number has been specified in the figure legends. Analysis was performed using Prism software (GraphPad Software), MedCalc statistical software version 18.9, and TIBCO Spotfire software (TIBCO Software).

## Results

### Subject Demographics

One hundred ten patients with IPF and 11 control subjects were enrolled in the present study. Demographic and clinicopathological features are shown in Table 1. Healthy volunteers had no self-reported history of lung disease, an absence of infection within the last 6 months, and normal spirometry. FVC percent predicted was significantly higher in healthy control subjects than in patients with IPF (*P* < 0.05, Mann-Whitney *U* test).

**Table 1.** Clinical Characteristics of Patients Included in This Study

	Healthy	IPF (Discovery)	IPF (Validation)
Number	11	97	13
Age, yr, mean (min–max)	40 (22–65)	70 (52–91)	68 (38–78)
Male sex, *n* (%)	7 (63%)	71 (73%)	6 (46%)
Smoking (ever vs. never), *n* (%)	4 (36%T)	61 (63%)	9 (69%)
FVC, % (±SD)	102.4 (±14.5)	74.56 (±15.2)	92.14 (±17.0)
Dl_CO_, % (±SD)	NA	46.88 (±13.2)	54.45 (±17.31)
Total cells in BAL, ×10^6^/ml, mean (±SD)	0.126 (±0.07)	0.268 (±0.23)	0.383 (±0.18)

*Definition of abbreviations*: IPF = idiopathic pulmonary fibrosis; max = maximum; min = minimum; NA = not applicable.

### Proportions of CD71^−^ AMs Are Increased in IPF BAL Compared with Healthy Controls

**Figure 1.**
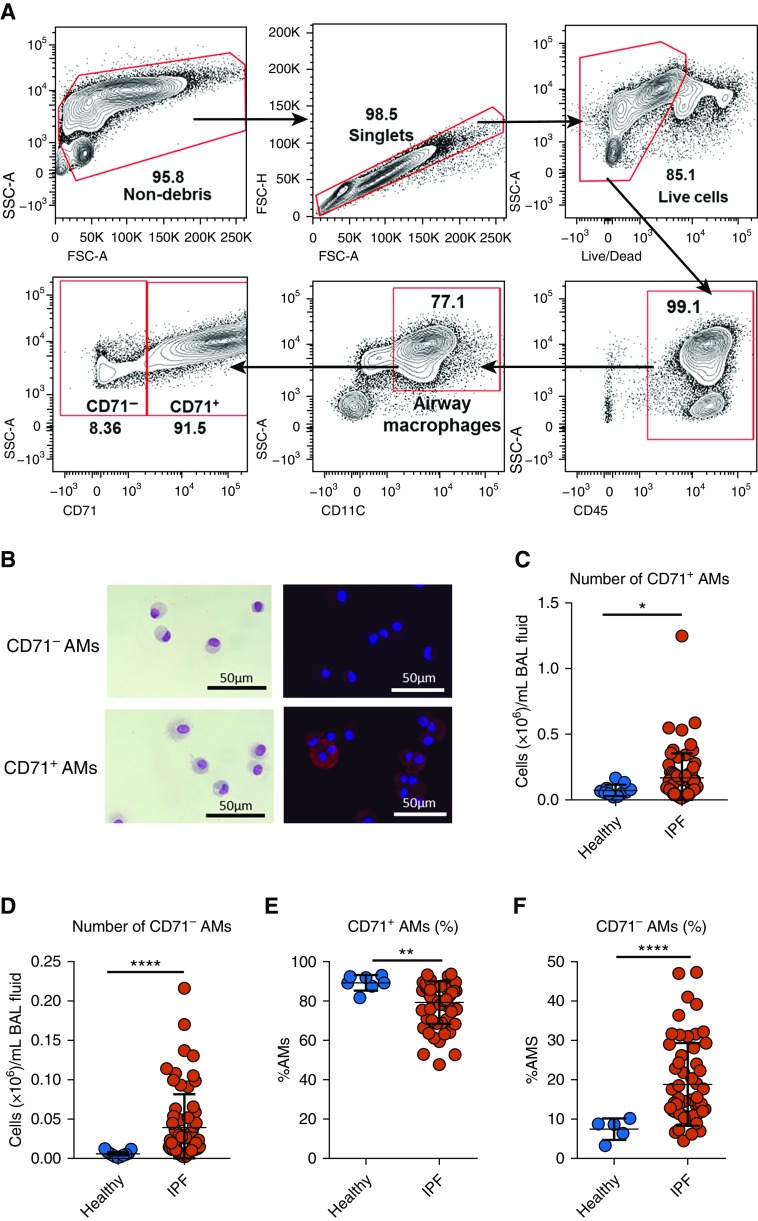
Proportions of CD71^−^ airway macrophages (AMs) are increased in idiopathic pulmonary fibrosis (IPF) BAL compared with healthy controls. (*A*) Gating strategy for flow cytometric analysis of BAL CD71-expressing AMs. (*B*) Wright-Giemsa staining of sorted CD71^−^ AMs (upper left) and CD71^+^ AMs (lower left) and immunofluorescence staining for CD71 in sorted CD71^−^ AMs (upper right) and CD71^+^ AMs (lower right). (*C* and *D*) Numbers of CD71^+^ (*C*) and CD71^−^ (*D*) AMs in healthy and IPF BAL. (*E* and *F*) Proportions of CD71^+^ (*E*) and CD71^−^ (*F*) AMs in healthy and IPF BAL. *n* = 11 healthy controls; *n* = 97 IPF. Data are presented as mean ± SD. *P < 0.05, **P < 0.01, and ****P < 0.0001, Mann-Whitney *U* test. FSC = forward scatter; SSC = side scatter.

To define whether proportions of CD71-expressing AMs were altered during IPF, we used a multicolor flow cytometry gating strategy. After exclusion of debris and doublets and selection of live cells, AMs were defined as CD45^+^ cells expressing CD11c (Figure 1A). In this gate, two clear AM populations were apparent, distinguishable by the expression of CD71 (Figure 1A), based on fluorescence-minus-one and isotype controls (Figure E1A in the online supplement). Because dendritic cells and monocytes may also be CD45^+^CD11c^+^, we performed additional analysis to assess potential contamination within the AM gate; the gating strategy employed is outlined in Figure E1B. After gating on CD45^+^CD11c^+^SSC^hi^ cells, we selected cells negative for lineage markers (CD3, CD4, CD8, CD19, CD20, CD34, FcεRI) and positive for HLA-DR^+^. Lin^−^HLA-DR^+^ cells were separated into CD14^+^CD16^−^ classical monocytes, CD14^+^CD16^+^ intermediate monocytes, CD14^−^CD16^+^ nonclassical monocytes, HLA-DR^+^CD11C^+^ myeloid-derived DCs, and CD11c^−^CD123^+^ plasmacytoid DCs, and each subtype was expressed as a proportion of CD45^+^CD11c^+^SSc^hi^ cells. Lin^−^CD11c^+^ cells accounted for around 1–2% of the AM gate, and DCs and monocytes comprised a minor population within these (Figure E1C). Our AM gating strategy was further confirmed by examination of cytospins of sorted CD71^+^ and CD71^−^ AMs (Figure 1B). Immunohistochemical staining of cytospins confirmed the expression of CD71 on AMs from the CD71^+^ gate and a lack of staining on those sorted from the CD71^−^ gate (Figure 1B). The CD71^−^ AM population was smaller and less granular than CD71^+^ AMs (Figure E1D). Numbers of both CD71^+^ and CD71^−^ AMs were increased in IPF BAL compared with healthy controls (Figures 1C and 1D). Interestingly, the proportions of CD71^+^ AMs cells were significantly decreased in IPF BAL compared with healthy controls (Figure 1E), with a concomitant increase in CD71^−^ AMs during fibrotic lung disease (Figure 1F). We confirmed these findings in a second validation cohort of 13 patients with IPF (Figures E1E–E1H). When we separated control subjects into younger (22–48 yr) or older subjects (>49 yr), we found no significant alteration in the proportions of CD71^+^ (Figure E1I) or CD71^−^ (Figure E1J) AMs. However, there was a decrease in absolute numbers of both CD71^+^ (Figure E1K) and CD71^−^ AMs (Figure E1L) when we compared aged with younger healthy control subjects. We further confirmed our findings by using an alternate gating strategy to define AMs. CD206 has been shown to be highly expressed in AM populations (22, 24); pregating on CD11c^+^CD206^+^ AMs and subsequently on CD71^+/−^ AMs (Figure E2A) recapitulated the results shown in Figures 1C–1F and E1 (Figures E2B–E2I). In addition, we performed an analysis of both innate and adaptive immune cell populations in the IPF BAL (Figure E2J). Together, these data indicate that during homeostasis, the majority of AMs express CD71; however, in the IPF lung, there is a distinct expansion of AM populations lacking the transferrin receptor.

### Defective Transferrin Uptake in CD71^−^ AMs

**Figure 2.**
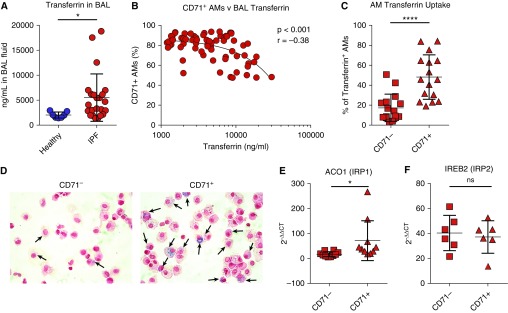
Defective transferrin uptake in CD71^−^ airway macrophages (AMs). (*A*) Concentrations of transferrin in the BAL of patients with idiopathic pulmonary fibrosis (IPF) or of healthy control subjects (*n* = 7 healthy control subjects; *n* = 31 patients with IPF). (*B*) Correlation of proportions of BAL CD71^+^ AMs with concentrations of transferrin in the airways (*n* = 67 patients with IPF). (*C*) Comparison of transferrin uptake in IPF-CD71^−^ and IPF-CD71^+^ AMs. (*D*) Staining (Prussian blue) for iron in sorted CD71^−^ or CD71^+^ AMs. Arrows indicate iron-positive AMs. (*E* and *F*) Gene expression analysis of sorted CD71^−^ or CD71^+^ AMs is shown for ACO1 (*n* = 10 patients with IPF) (*E*) and IREB2 (*n* = 6 patients with IPF) (*F*). *GAPDH* was used as a housekeeping gene. Data are presented as mean ± SD. *P < 0.05 and ****P < 0.0001, Mann-Whitney *U* test. ns = not significant.

Because IPF AMs were marked by a lack of the transferrin receptor CD71, we next assessed the concentrations of transferrin in the airways and the ability of IPF CD71^+/−^ AMs to sequester this complex. Significantly higher concentrations of transferrin were detected in IPF BAL than in healthy controls (Figure 2A). There was an inverse correlation between the concentrations of transferrin in the airways compared with the proportion of AMs expressing CD71 (Figure 2B). Next, using flow cytometry, we examined the ability of the cells to take up transferrin. Our analysis revealed a significant increase in transferrin uptake in the CD71^+^ cells compared with AMs lacking CD71 (Figure 2C). Free iron concentrations (without prior acid digestion) were also examined but were not detected in IPF or healthy BAL supernatants (data not shown); however, we detected iron-containing AMs by Prussian blue staining of cytospin preparations (Figure 2D), and CD71^+^ AMs appeared to sequester more iron than their CD71^−^ counterparts. We next examined the expression of genes involved in iron homeostasis in CD71^+/−^ AMs derived from patients with IPF. IRP1 (iron regulatory protein 1), which binds and stabilizes CD71 mRNA (25), was overexpressed in CD71^+^ compared with CD71^−^ AMs (Figure 2E), whereas IRP2 showed similar expression levels between both cells (Figure 2F). Additional genes involved in iron homeostasis were examined, including HMOX1 (heme oxygenase [decycling] 1), ferroportin (SLC40A1), and HAMP, revealing no significant differences in expression of these factors compared with CD71-expressing AMs (Figures E3A–E3C).

### CD71^+^ AMs Are Phenotypically Distinct from CD71^−^ AMs

**Figure 3.**
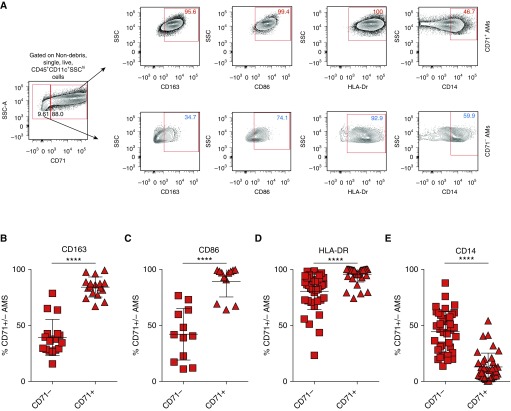
CD71^+^ and CD71^−^ airway macrophages (AMs) are phenotypically distinct. (*A*) Flow cytometric gating strategy for the analysis of AM phenotypic markers. Data are shown as a percentage of either CD71^+^ AMs expressing a marker of interest (highlighted in red) or CD71^−^ AMs expressing a marker of interest (highlighted in blue). (*B*–*E*) Proportions of idiopathic pulmonary fibrosis (IPF)-CD71^+^ and IPF-CD71^−^ AMs expressing CD163 (*n* = 17 patients with IPF) (*B*), CD86 (*n* = 12 patients with IPF) (*C*), HLA-DR (*n* = 38 patients with IPF) (*D*), and CD14 (*n* = 38 patients with IPF) (*E*). Data are presented as mean ± SD. ****P < 0.0001 by Mann-Whitney *U* test. SSC = side scatter.

Because CD71^−^ AMs were elevated in IPF and demonstrate defective iron-sequestering abilities, and because iron status is known to influence macrophage activation (26), we next contrasted the expression of macrophage phenotypic markers in CD71^+/−^ AM populations. Cells were pregated on either SSc^hi^CD45^+^CD11c^+^CD71^+^ or SSc^hi^CD45^+^CD11c^+^CD71^−^ cells, and subsequently, we assessed the proportion of CD71^+^ or CD71^−^ AMs that were positive for the marker of interest (Figure 3A). We found that multiple cell surface proteins associated with macrophage activation were expressed at higher levels in CD71^+^ AMs compared with the CD71^−^ population. These included the high-affinity scavenger receptor for the Hb–haptoglobin complex CD163 (Figure 3B) and CD86 (Figure 3C). Of note, HLA-DR has been proposed to identify mature resident AMs in the airways (22, 27) and was found to be elevated in CD71^+^ AMs (Figure 3D); conversely, the monocyte marker CD14 was significantly increased in CD71^−^ AMs compared with CD71^+^ AMs (Figure 3E).

### CD71 Delineates Functionally Distinct AM Populations

**Figure 4.**
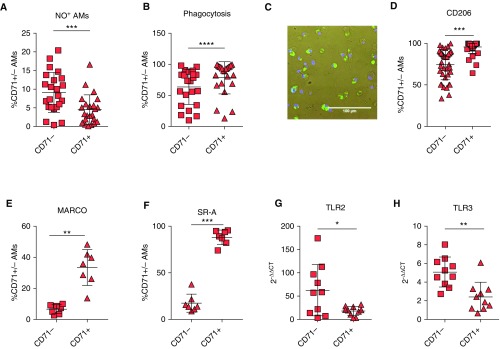
CD71^+^ and CD71^−^ airway macrophages (AMs) are functionally distinct. (*A*) Comparison of nitric oxide (NO) expression in idiopathic pulmonary fibrosis (IPF)-CD71^+^ and IPF-CD71^−^ AMs (*n* = 23 patients with IPF). (*B*) Phagocytosis assay of IPF-CD71^+^ and IPF-CD71^−^ AMs (*n* = 23 patients with IPF). (*C*) Representative staining of AMs after phagocytosis assay showing phagocytosed bacteria (green) and DAPI (blue). (*D–F*) Proportions of IPF-CD71^+^ and IPF-CD71^−^ AMs expressing CD206 (*n* = 39 patients with IPF) (*D*), MARCO (*n* = 7 patients with IPF) (*E*), and SR-A (*n* = 7 patients with IPF) (*F*). (*G* and *H*) Expression of TLR2 (*n* = 10 patients with IPF) (*G*) and TLR3 (*n* = 10 patients with IPF) (*H*) in CD71^+/−^ AMs. *GAPDH* was used as a housekeeping gene. Data are presented as mean ± SD. *P < 0.05, **P < 0.01, ***P < 0.001, and ****P < 0.0001, Mann-Whitney *U* test.

AMs play an important role in the detection and elimination of pathogens, and defects in iron sequestration may permit inappropriate pulmonary bacterial growth. Because the microbiome in patients with IPF has been reported to be altered (28), and because increased bacterial burden correlates with clinical decline (29), we next examined the ability of CD71^+/−^ AMs to detect and eliminate pathogens. Assessment of unstimulated cells revealed a significant increase in the presence of nitric oxide in CD71^−^ cells compared with CD71^+^ (Figure 4A). In addition, the ability of CD71^+/−^ cells to phagocytose bacteria (*S. aureus*) was assessed; CD71^+^ displayed an enhanced phagocytosis ability compared with the CD71^−^ AMs (Figures 4B and 4C). We assessed the expression of several receptors known to be involved in AM phagocytosis of *S. aureus*; CD206 (also known as the mannose receptor) has been implicated in the phagocytic uptake of pathogens (30). Macrophage scavenger receptors, such as MARCO (also known as SR-A6) and CD204 or SR-A, have been shown to play a key role in AM phagocytosis (31, 32). Percentages of AMs expressing CD206 (Figure 4D), MARCO (Figure 4E), and SR-A (Figure 4F) were increased in CD71^+^ AMs compared with CD71^−^ AMs. Analysis of genes involved in phagocytosis were also quantified. ELMO, which is involved in antibody-mediated phagocytosis, and FCγRIB did not differ in CD71^+/−^ AMs (Figures E4A and E4B). TLRs are critical in the detection of pathogen-associated molecular patterns and damage-associated molecular patterns. The expression of TLR genes was examined in CD71^−^ and CD71^+^ cells. Expression of TLR2 (Figure 4G) and TLR3 (Figure 4H), but not of TLR1, 4, 6, 7, or 9 (Figures E4C–E4G), was significantly increased in CD71^−^ AMs compared with CD71^+^.

### CD71^−^ AMs Have Profibrotic Characteristics

**Figure 5.**
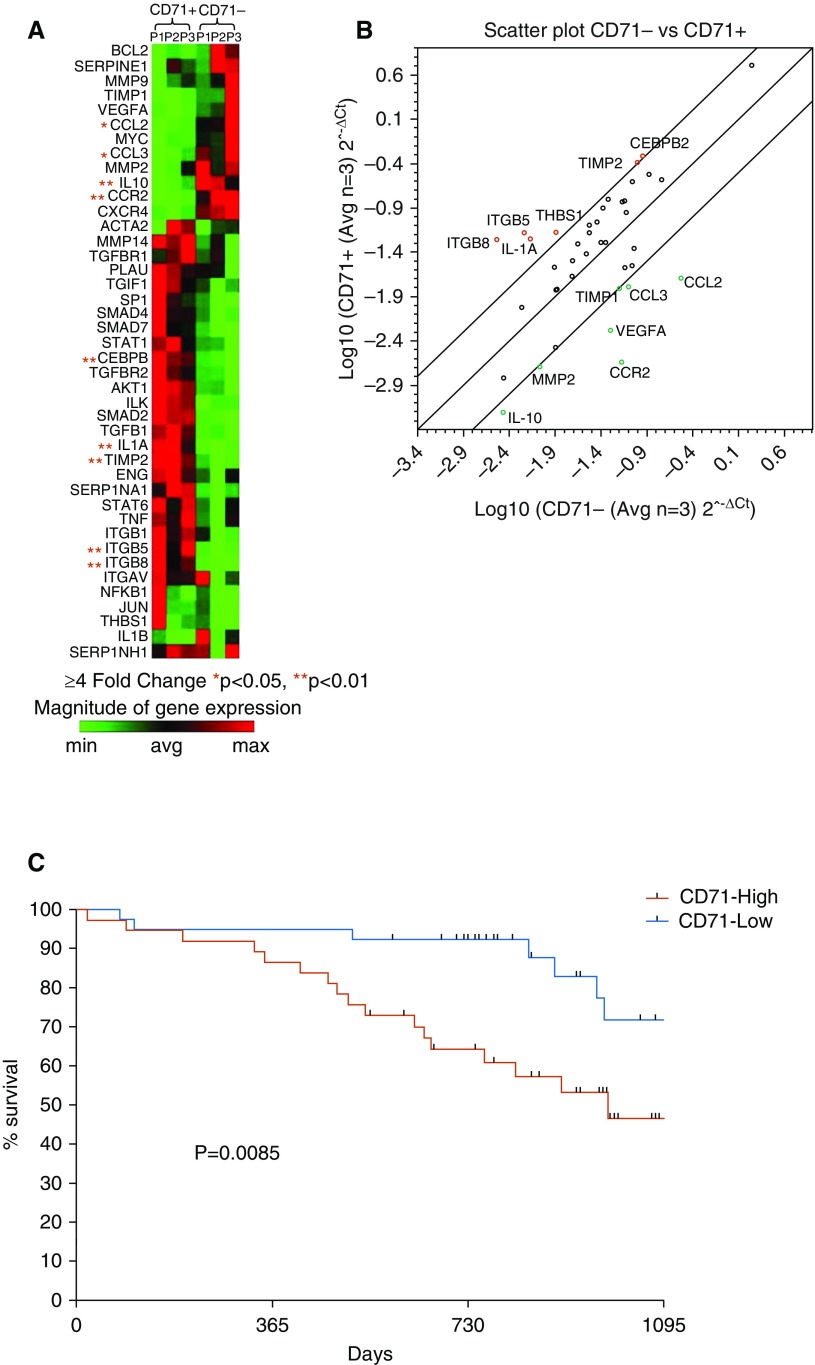
CD71^−^ airway macrophages (AMs) have profibrotic characteristics. (*A*) Heat map representation of fibrosis array of sorted CD71^+/−^ idiopathic pulmonary fibrosis (IPF)-AMs. (*B*) Scatterplot highlighting differentially expressed genes in CD71^+^ and CD71^−^ IPF-AMs. Plot shows fibrosis-related genes upregulated in CD71^−^ AMs compared with CD71^+^ cells in green and those downregulated in CD71^−^ AMs compared with CD71^+^ AMs in red. Genes with statistically significant and greater than twofold change compared with control are highlighted. (*C*) Kaplan-Meier survival plot for subjects with IPF dichotomized above (High) and below (Low) the median for proportion of CD71^−^ AMs. Individuals with an increased proportion of CD71^−^ AMs have worse survival than patients with a low proportion of CD71^−^ AMs (*n* = 97 patients with IPF).

To further examine the role of CD71-expressing AMs during IPF, we used a PCR array that interrogates 84 genes involved in the remodeling cascade (Figures 5A and 5B). Figure 5A highlights significantly differentially expressed genes, showing CCL2, CCL3, IL-10, and CCR2 to be significantly increased in CD71^−^ cells, whereas CEBPB (CCAAT/enhancer-binding protein-β), IL1A, TIMP2 (metalloproteinase inhibitor 2), and ITGB5 and ITGB8 (integrins-β5 and -β8, respectively) were increased in the CD71^+^ cells. Figure 5B highlights genes that showed at least a fourfold increase in CD71^−^ compared with CD71^+^ cells. Fibrosis-related genes upregulated in CD71^−^ AMs compared with CD71^+^ cells are shown in green, and those downregulated in CD71^−^ AMs compared with CD71^+^ AMs are shown in red. To assess the clinical impact of CD71^−^ status, we next asked whether the percentage of CD71^−^ AMs was related to survival in patients with IPF. Kaplan-Meier survival analyses showed a significant relationship (*P* = 0.0085) between CD71 status and survival when subjects were dichotomized to those above and below the median (Figure 5C). There was no correlation between proportions of CD71^−^ AMs and baseline disease severity (measured by FVC and Dl_CO_), suggesting that CD71^−^ AM status is an independent determinant of outcome in IPF. In a Cox proportional hazards model, both baseline FVC and percentage CD71^−^ AMs were associated with survival, with a hazard ratio of 0.9639 (95% confidence interval, 0.9317–0.9972; *P* = 0.0338) and 1.0372 (95% confidence interval, 1.0137–1.0612; *P* = 0.0018), for subjects with IPF dichotomized above and below the median for proportion of CD71^−^ AMs, respectively.

## Discussion

AMs are key components of lung immunity and provide the first line of defense against inhaled particulates and invading pathogens. Although iron is required for normal cellular functions, inappropriately high concentrations may accumulate in the lung through inhaling iron-containing particulate matter such as pollutants, cigarette smoke, or siderophores (19, 33). It is therefore critical that AMs are capable of efficiently sequestering and processing excess iron in the lung without inappropriate activation. Our data indicate that during IPF, there is an expansion of AMs, which are defined by defective transferrin uptake, functional and phenotypic immaturity, and expression of profibrotic factors. Together, our data identify a novel population of AMs that mark disease progression during IPF and identify the CD71 pathway as a potential target for therapeutic intervention during fibrosing lung disease.

Multiple recent reports have indicated that lung-resident AMs maintain their populations via proliferation *in situ* during homeostasis (34–37) and also that, during ongoing inflammatory responses, monocytes are recruited to the lung and develop into AM-like cells (36, 38). Misharin and colleagues recently showed that monocyte-derived rather than fetally derived tissue-resident AMs were essential for the development of pulmonary fibrosis in murine models, whereas deletion of tissue-resident AMs had no effect on the disease (39). However, little is known regarding AM functional subsets in the human lung and, indeed, during IPF. CD71 may delineate a population of protective resident AMs, whereas monocyte-derived AMs are defined by a lack of CD71^−^ expression and drive pathology during IPF, analogous to AMs found in murine models of fibrosing lung disease (13). CD71 has been described as a marker of mature macrophages (40, 41), with increased expression reported in AMs in a murine model of fibrosis (42), in addition to increased expression on ILD AMs (21). We found enhanced expression of multiple markers that have been described as markers of mature AM phenotypes, such as HLA-DR and CD206 on CD71^+^ AMs compared with the CD71^−^ AM population. Conversely, CD71^−^ AMs were defined by high expression of the classical monocyte activation marker CD14, in addition to expression of CCL2 and its receptor CCR2, compared with the CD71^+^ population. Similarly, there was an increase in the expression of chemokine receptor CXCR4 and chemokine CCL3 (MIP-1α) in CD71^−^ cells. Examination of morphology of AMs from patients with IPF indicated that CD71^−^ cells were smaller and less granular than CD71^+^ AMs. Taken together, this suggests that CD71^−^ cells may have been recruited into the lung and may have played a role in further recruitment of monocyte-derived cells. However, we cannot discount the possibility that the local environmental milieu during IPF leads to the downregulation of CD71 in tissue-resident AMs.

Interestingly, multiple fibrotic pathways appeared to be activated in CD71^−^ AMs. Of note, expression of MMP2 and MMP9 was increased in CD71^−^ cells compared with CD71^+^ AMs, suggesting a role in transforming growth factor-β activation in the fibrotic lung (4). Additional remodeling genes were increased in CD71^−^ cells, including *VEGFA* (vascular endothelial growth factor A) and *SERPINE1* (serine protease inhibitor family E member 1) encoding PAI-1 (plasminogen activator inhibitor 1). PAI-1 inhibits the enzyme uPA (urokinase plasminogen activator), which is responsible for the cleavage of plasminogen to form plasmin, which in turn mediates the degradation of the ECM (43). The transcription factor MYC was increased in CD71^−^ cells. MYC plays a key role in cell cycle progression and has been shown to be critical in differentiating monocytes into osteoclasts, a specialized bone tissue macrophage, suggesting a possible role for MYC differentiation in CD71^−^ cells (44). BCL2 (B-cell lymphoma 2) is also increased in CD71^−^ cells, which plays a role exerting antiapoptotic effects (45).

Iron sequestration by AMs represents a protective innate immune mechanism that limits available free iron concentrations, which are used by pathogenic microorganisms. Recent work has indicated that there is a significant difference between the microbiota in subjects with IPF and healthy control subjects (28) and, furthermore, that alterations in the pulmonary bacterial burden are related to exacerbations of the disease (29, 46). The observed differences in TLR expression, nitric oxide production, phagocytotic ability, and transferrin uptake in CD71^+/−^ populations suggest a discordant ability to eliminate infectious agents in these populations. Although CD71 AMs have impaired transferrin uptake, and although there is an inverse correlation between the concentrations of transferrin in the airways and the proportion of AMs expressing CD71, it should be noted that there were numerically more CD71^+^ AMs in the BAL of patients with IPF than in healthy individuals. It is therefore likely that other mechanisms contribute to the increased transferrin load observed in IPF, including increased vascular leak and exposure of airway cells to Hb as well as synthesis of transferrin by immune or stromal cells (such as airway epithelial cells). Alternative phagocytosis mechanisms, such as mannose detection–linked phagocytosis and increased scavenger receptor expression (MARCO and SR-A), may explain why a greater proportion of CD71^+^ cells phagocytosed bacteria, because they expressed significantly more mannose receptor than CD71^−^ cells. It is of interest that iron could be readily detected in AMs but that there were minimal concentrations in lavage supernatants (without prior acid treatment). These results are perhaps not surprising, given that free iron is both an important cofactor required for replication by many species of pathogenic bacteria and a potent oxidizing agent. Non–protein-bound iron–clearing mechanisms in all tissues of the body, including binding by transferrin and phagocytosis by macrophages, are therefore rapid, highly efficient, and tightly regulated. In the lung, the majority of residual free iron is likely sequestered by bacteria, resulting in unmeasurable concentrations and reflecting the importance of iron clearance mechanisms in the lung.

In summary, our findings indicate that during IPF, there is an expansion of a population of AMs with impaired transferrin receptor expression and defects in components of free iron uptake and bacterial phagocytosis. Our work is of clinical relevance because proportions of CD71^−^ AMs are expanded in patients with progressive fibrotic disease compared with those presenting with a nonprogressive disease course. These data suggest that iron metabolism and CD71-expressing AMs may be both a novel marker of poor prognosis during IPF and a potential therapeutic target.

## Supplementary Material

Supplements

Author disclosures
