# Supplementary material for: The Transferrin Receptor CD71 Delineates Functionally Distinct Airway Macrophage Subsets during Idiopathic Pulmonary Fibrosis
Source: Am J Respir Crit Care Med. 2019 Jul 15;200(2):209–19. doi: 10.1164/rccm.201809-1775OC (PMC6635794; doi:10.1164/rccm.201809-1775OC)
Supplement: Supplements [file rccm.201809-1775OC.html]

The Transferrin Receptor CD71 Delineates Functionally Distinct Airway Macrophage Subsets during Idiopathic Pulmonary Fibrosis | American Journal of Respiratory and Critical Care Medicine

- disclosures.pdf (217 KB)
- allden\_data\_supplement.pdf (766 KB)
