# Supplementary material for: The Transferrin Receptor CD71 Delineates Functionally Distinct Airway Macrophage Subsets during Idiopathic Pulmonary Fibrosis
Source: Am J Respir Crit Care Med. 2019 Jul 15;200(2):209–19. doi: 10.1164/rccm.201809-1775OC (PMC6635794; doi:10.1164/rccm.201809-1775OC)
Supplement: Supplements [file rccm.201809-1775OC_allden_data_supplement.pdf]

The transferrin receptor CD71 delineates functionally distinct airway macrophage subsets during idiopathic pulmonary fibrosis

ONLINE DATA SUPPLEMENT

Supplementary Figure E1. **A) FMO controls for CD71 stain. B)** Gating strategy for identification of BAL monocytes or DCs by polychromatic flow cytometry. Proportions of lineage negative cells (Lin^-), classical (CMs), intermediate (IMs), non-classical (NCMs) monocytes, or total DCs in the $CD45^+CD11c^+Ssc^{hi}$ gate are shown in (n=14 IPF patients, **C**). **D**) Back gating of $CD71^+$ (indicated in red) and $CD71^-$ (indicated in blue) AMs. Numbers of $CD71^+$ (**E**) and $CD71^-$ (**F)** AMs in a validation cohort (n=11 healthy controls, n=13 IPF). Proportions of $CD71^+$ (**G**) and $CD71^-$ (**H**) AMs in healthy and IPF BAL in a validation cohort. Proportions of $CD71^+$ (**I**) and $CD71^-$ (**J)** AMs in younger and older healthy controls. Numbers of $CD71^+$ (**K**) and $CD71^-$ (**L)** AMs in younger and older healthy controls (n=7 aged 22-48 y.o., n=4 >49 y.o.). Data presented as mean plus standard deviation; *$p<0.05$, ***$p<0.001$ ****$p<0.0001$, Mann-Whitney U test.

Supplementary Figure E2. A) Alternate gating strategy for the identification of CD71-expressing AMs. Numbers of $CD71^+$ (**B**) and $CD71^-$ (**C)** $CD206^+CD11c^+$ AMs in healthy and IPF BAL in the discovery cohort. Proportions of $CD71^+$ (**D**) and $CD71^-$ (**E**) $CD206^+CD11c^+$ AMs in healthy and IPF BAL in the discovery cohort. Numbers of $CD71^+$ (**F**) and $CD71^-$ (**G)** CD206 $^+CD11c^+$ AMs in healthy and IPF BAL in the validation cohort. Proportions of $CD71^+$ (**H**) and $CD71^-$ (**I**) $CD206^+CD11c^+$ AMs in healthy and IPF BAL in the validation cohort. **J**) Gating strategy for the identification of the major immune cell types in IPF BAL, including airway macrophage (AMs), granulocytes (Gran), Lymphocytes (Lymph), neutrophils (NΦ), classical monocytes (CMs), eosinophils (Eos), mast cells, natural killer (NK) cells, natural killer-T (NKT) cells, T- and B-cells. Data presented as mean plus standard deviation; **$p<0.01$, ***$p<0.001$ ****$p<0.0001$, Mann-Whitney U test.

Supplementary Figure E3: Expression of HMOX (n=10 IPF patients, **A**), SLC40A1 (n=10 IPF patients, **B**) and HAMP (n=10 IPF patients, **C**), in $CD71^{+/-}$ AMs. Data presented as mean and standard deviation.

Supplementary Figure E4. Expression of ELMO (n=5 IPF patients, **A**), FCγR1B (n=9 IPF patients, **B**), TLR1 (n=8 IPF patients, **C**), TLR4 (n=6 IPF patients, **D**), TLR6 (n=8 IPF patients, **E**), TLR1 (n=8 IPF patients, **F**), TLR7 (n=8 IPF patients, **F**) and TLR9 (n=5 IPF patients, **G**) in $CD71^{+/-}$ AMs. GAPDH was used as housekeeping gene, data is presented as mean and standard deviation.

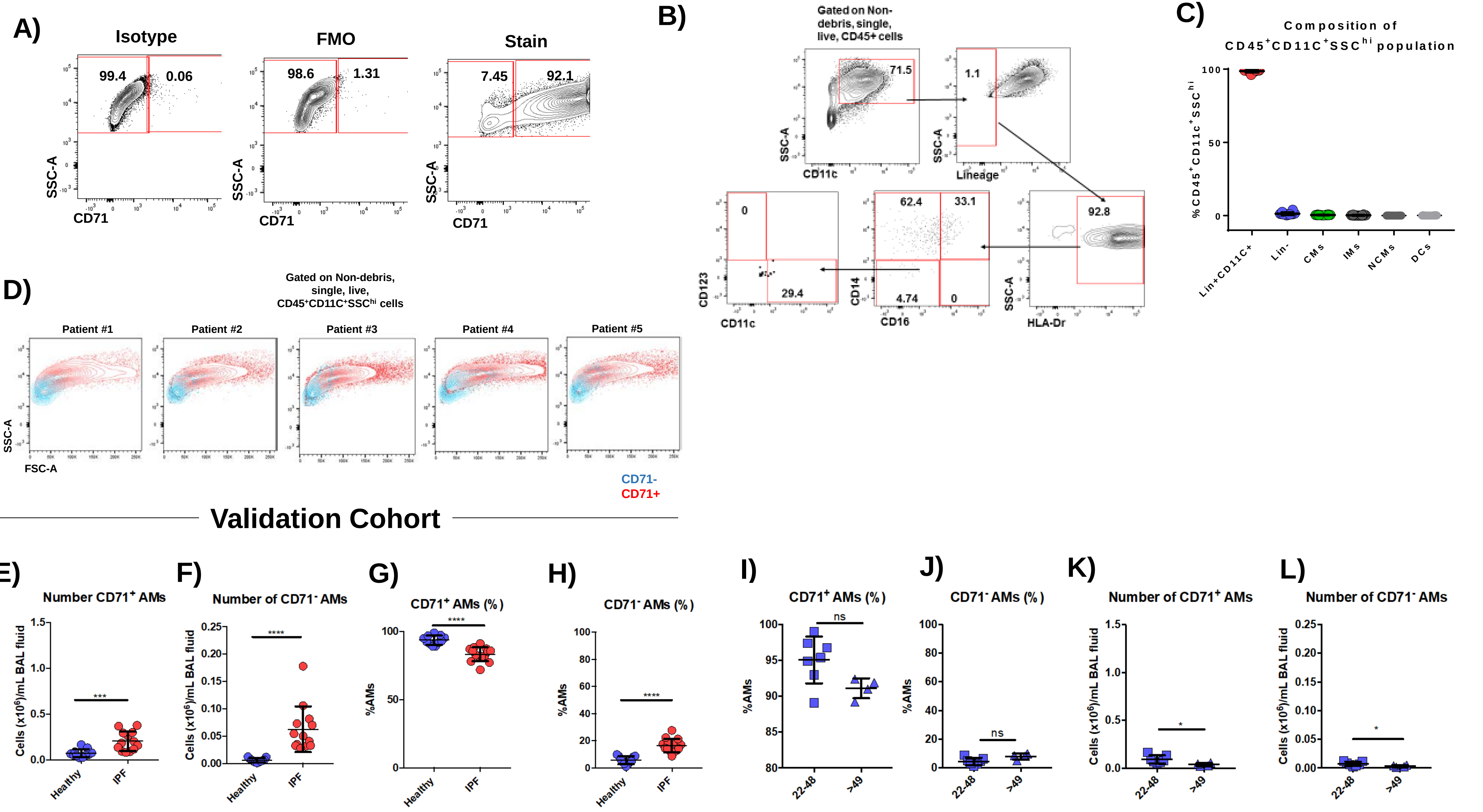
A)
Isotype
FMO
Stain
99.4
0.06
98.6
1.31
7.45
92.1
SSC-A
CD71
B)
Gated on Non-debris, single, live, CD45+ cells
71.5
1.1
SSC-A
CD11c
Lineage
92.8
HLA-Dr
62.4
33.1
4.74
0
CD14
CD16
0
29.4
CD123
CD11c
C)
Composition of CD45+CD11C+SSChi population
%CD45+CD11c+SSChi
Lin+CD11C+
Lin-
CMs
IMs
NCMs
DCs
D)
Gated on Non-debris, single, live, CD45+CD11C+SSChi cells
Patient #1
Patient #2
Patient #3
Patient #4
Patient #5
SSC-A
FSC-A
CD71-
CD71+
Validation Cohort
E)
Number CD71+ AMs
Cells (x10^6)/mL BAL fluid
***
Healthy
IPF
F)
Number of CD71- AMs
Cells (x10^6)/mL BAL fluid
****
Healthy
IPF
G)
CD71+ AMs (%)
%AMs
****
Healthy
IPF
H)
CD71- AMs (%)
%AMs
****
Healthy
IPF
I)
CD71+ AMs (%)
%AMs
ns
22-48
>49
J)
CD71- AMs (%)
%AMs
ns
22-48
>49
K)
Number of CD71+ AMs
Cells (x10^6)/mL BAL fluid
*
22-48
>49
L)
Number of CD71- AMs
Cells (x10^6)/mL BAL fluid
*
22-48
>49

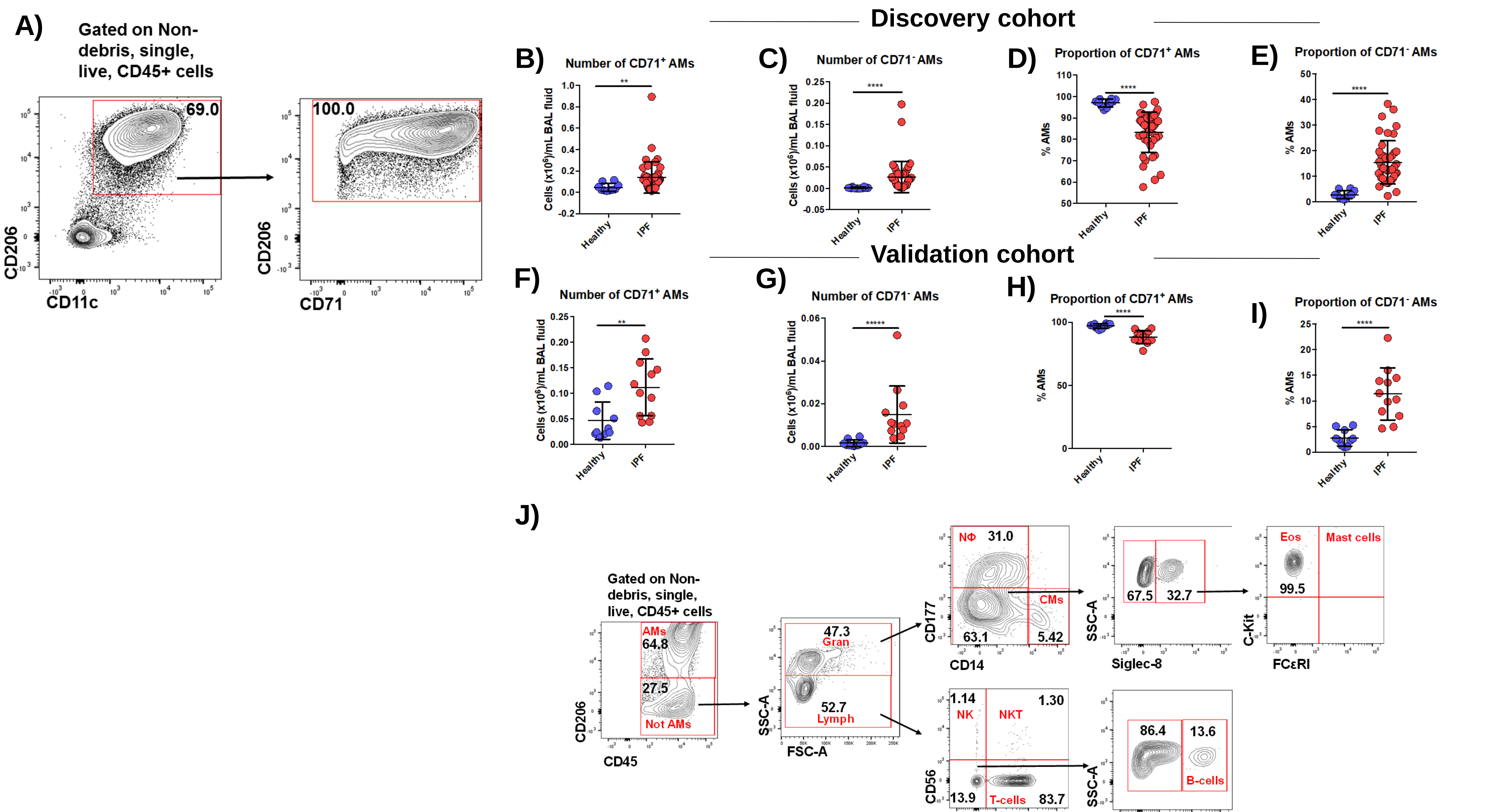
Discovery cohort
Validation cohort
A)
Gated on Non-debris, single, live, CD45+ cells
CD206
CD11c
69.0
CD71
100.0
B) Number of CD71+ AMs
C) Number of CD71- AMs
D) Proportion of CD71+ AMs
E) Proportion of CD71- AMs
F) Number of CD71+ AMs
G) Number of CD71- AMs
H) Proportion of CD71+ AMs
I) Proportion of CD71- AMs
Cells (x10⁶)/mL BAL fluid
% AMs
Healthy
IPF
J)
AMs
64.8
27.5
Not AMs
CD45
47.3
Gran
52.7
Lymph
SSC-A
FSC-A
NΦ
31.0
CMs
63.1
5.42
CD177
CD14
67.5
32.7
Siglec-8
Eos
Mast cells
99.5
C-Kit
FCεRI
1.14
NK
NKT
1.30
13.9
T-cells
83.7
CD56
CD3
86.4
13.6
B-cells
CD19

Figure E2

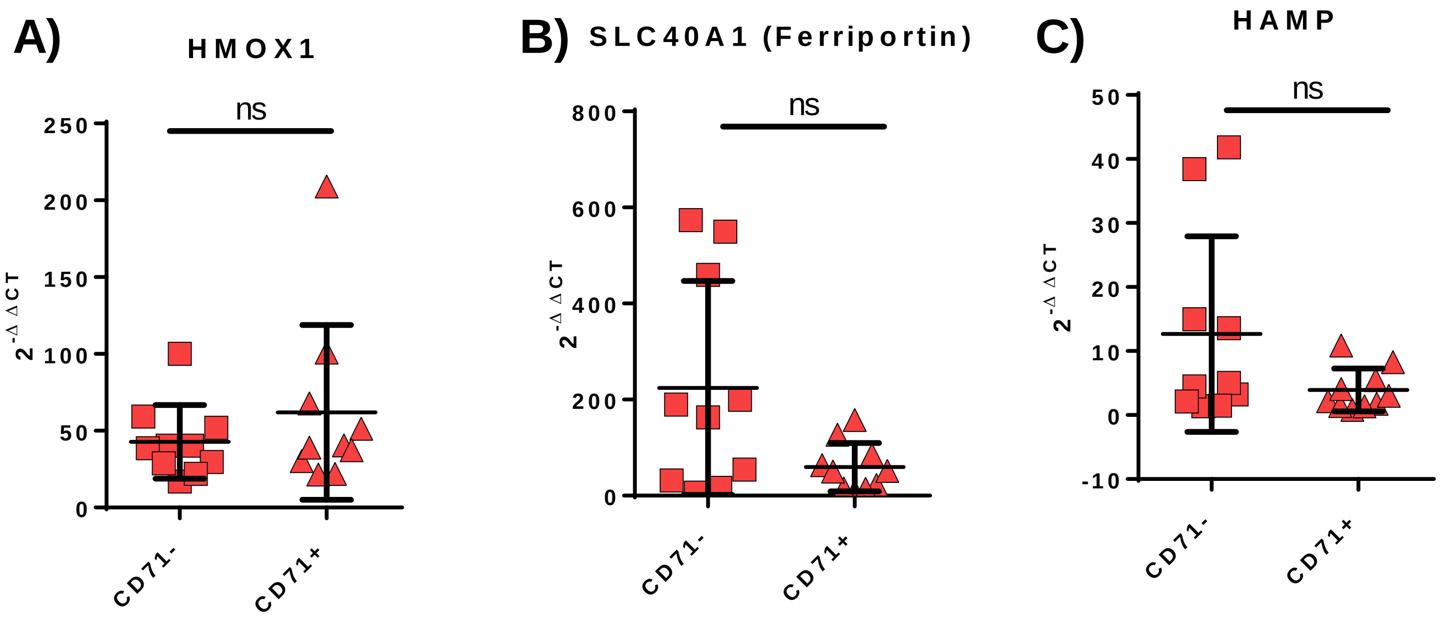

A)
HMOX1
ns
250
200
150
100
50
0
2-ΔΔCT
CD71-
CD71+
B)
SLC40A1 (Ferriportin)
ns
800
600
400
200
0
2-ΔΔCT
CD71-
CD71+
C)
HAMP
ns
50
40
30
20
10
0
-10
2-ΔΔCT
CD71-
CD71+

Figure E3

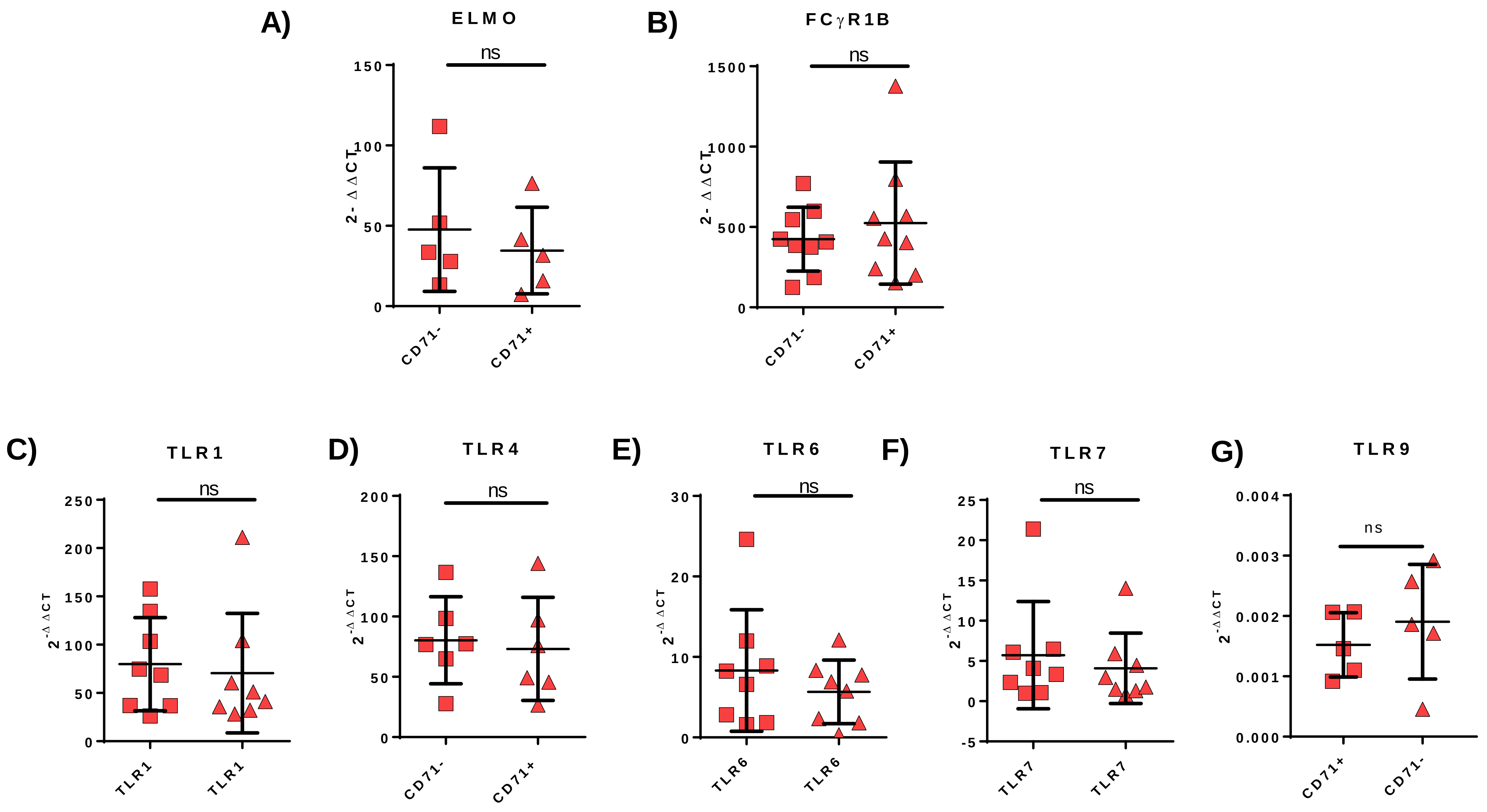

A)
ELMO
ns
2-ΔΔCT
150
100
50
0
CD71-
CD71+
B)
FCγR1B
ns
2-ΔΔCT
1500
1000
500
0
CD71-
CD71+
C)
TLR1
ns
2-ΔΔCT
250
200
150
100
50
0
TLR1
TLR1
D)
TLR4
ns
2-ΔΔCT
200
150
100
50
0
CD71-
CD71+
E)
TLR6
ns
2-ΔΔCT
30
20
10
0
TLR6
TLR6
F)
TLR7
ns
2-ΔΔCT
25
20
15
10
5
0
-5
TLR7
TLR7
G)
TLR9
ns
2-ΔΔCT
0.004
0.003
0.002
0.001
0.000
CD71+
CD71-

Figure E4
